# Individual Proportion Loss of Functional Connectivity Strength: A Novel Individual Functional Connectivity Biomarker for Subjective Cognitive Decline Populations

**DOI:** 10.3390/biology12040564

**Published:** 2023-04-07

**Authors:** Zhuoyuan Li, Hua Lin, Qi Zhang, Rong Shi, Huanyu Xu, Fan Yang, Xueyan Jiang, Luyao Wang, Ying Han, Jiehui Jiang

**Affiliations:** 1 School of Communication and Information Engineering, Shanghai University, Shanghai 200444, China; 2 Nuclear Medicine and Molecular Imaging Key Laboratory of Sichuan Province, Luzhou 646000, China; 3 Department of Neurology, Xuanwu Hospital of Capital Medical University, Beijing 100053, China; 4 Institute of Biomedical Engineering, School of Life Science, Shanghai University, Shanghai 200444, China; 5 School of Biomedical Engineering, Hainan University, Haikou 570228, China; 6 Center of Alzheimer’s Disease, Beijing Institute for Brain Disorders, Beijing 100053, China; 7 National Clinical Research Center for Geriatric Disorders, Beijing 100053, China

**Keywords:** subjective cognitive decline, Alzheimer’s disease, functional magnetic resonance imaging, individual proportion loss of functional connectivity strength

## Abstract

**Simple Summary:**

Objective biomarkers for the diagnosis of subjective cognitive decline (SCD) are critical for intervention in the Alzheimer’s disease (AD) disease process. Previous research has shown that functional magnetic resonance imaging (fMRI) is a significant tool for detecting abnormal interregional functional connectivity (FC) in SCD populations. However, traditional FC calculations do not reveal the high individual variation in the SCD population. We proposed a new framework for individual proportion loss of functional connectivity strength (IPLFCS) based on FC to identify SCD biomarkers, and further explore the relationship between biomarkers and amyloid deposition as well as neuropsychological performance. The study findings indicated that IPLFCS in the left middle temporal gyrus (LMTG) was identified as a potential biomarker that correlated with cortical amyloid deposition and cognitive performance.

**Abstract:**

High individual variation in the subjective cognitive decline (SCD) population makes functional connectivity (FC) biomarkers unstable. This study proposed a novel individual FC index, named individual proportion loss of functional connectivity strength (IPLFCS), and explored potential biomarkers for SCD using this new index. We proposed an IPLFCS analysis framework and compared it with traditional FC in Chinese and Western cohorts. Post hoc tests were used to determine biomarkers. Pearson’s correlation analysis was used to investigate the correlation between neuropsychological scores or cortical amyloid deposits and IPLFCS biomarkers. Receiver operating characteristic curves were utilized to evaluate the ability of potential biomarkers to distinguish between groups. IPLFCS of the left middle temporal gyrus (LMTG) was identified as a potential biomarker. The IPLFC was correlated with the traditional FC (r = 0.956, *p* < 0.001; r = 0.946, *p* < 0.001) and cortical amyloid deposition (r = −0.245, *p* = 0.029; r = −0.185, *p* = 0.048) in both cohorts. Furthermore, the IPLFCS decreased across the Alzheimer’s disease (AD) continuum. Its diagnostic efficiency was superior to that of existing fMRI biomarkers. These findings suggest that IPLFCS of the LMTG could be a potential biomarker of SCD.

## 1. Introduction

Alzheimer’s disease (AD) places a huge burden on society and families [1]. The progression of AD is irreversible, and it is generally believed that early intervention is of critical importance in managing the disease [2]. Recent studies have indicated that subjective cognitive decline (SCD) may represent an early asymptomatic stage in the clinical progression of AD [3]. SCD refers to the subjective perception of a decline in cognitive abilities, despite no clear cognitive impairment detected in neuropsychological testing [4]. The risk of developing AD is higher in individuals with SCD [5,6,7]. Therefore, the diagnosis of SCD with objective biomarkers can be effective in preventing its conversion to mild cognitive impairment (MCI) and AD and has an important role in the successful use of interventional therapies [8].

The functional magnetic resonance imaging (fMRI), as a major tool, has been widely used in the cognitive research. In our previous studies, it has been shown that machine learning verification using diffusion tensor imaging (DTI) and fMRI resulted in identification accuracies of approximately 60% and 76.44% for SCD, respectively [9]. By integrating fMRI and DTI modalities, the ability to distinguish SCD from NC increased to an accuracy of 80.24% [10]. Furthermore, our recent dual-center study found that hypometabolism of the right middle temporal gyrus could differentiate between the NC and SCD groups with an AUC of 0.638–0.717 [11]. These findings indicate that fMRI have the potential to serve as a more sensitive and powerful biomarker for SCD, particularly in populations where abnormal interregional functional connectivity (FC) has been detected through fMRI analysis [12,13,14]. Besides, the researchers observed a reduction in FC in the bilateral hippocampus of the SCD group in contrast to the normal control (NC) group [15]. The traditional FC within the default mode network showed decreasing trend across the normal, SCD, and MCI group [16]. Altered FCs is also related to global cortical amyloid deposition [17] and neuropsychological scores [18]. However, there is high individual variety in the SCD population. Traditional FC calculations based on group comparisons do not show individual variety [19,20,21]. Hence, an individual FC index was required.

Recently, studies have also attempted to accurately characterize FC strength, which contributes to the understanding of the neural underpinnings of cognitive abilities and behaviors [14,22,23,24]. In this process, physiological and non-physiological noises easily interfere with blood oxygenation level dependent (BOLD) signals in fMRI, such as respiration, blood pressure changes, and head movements [25,26,27]. These non-neuronal noises can interfere with fMRI analysis [28,29,30]. To solve this problem, researchers have proposed a novel metric related to the degree of FC weighting, called proportional loss of functional connection strength (PLFCS), which is less susceptible to fMRI signal-to-noise interference than traditional FC metrics and avoids bias due to the introduction of proportional thresholds [31,32]. The PLFCS incorporates the connection strength of normal controls for standardization and provides a good basis for eliminating individual differences. Cope et al. used PLFCS to calculate the average strength of connections in AD. The findings show that average FC and tau burden are negatively correlated, with average FC declining along with tau burden [33]. However, presenting an individual proportion loss of functional connectivity strength (IPLFCS) remains challenging. The relationship between PLFCS and pathological molecular mechanisms as well as clinical manifestations at the SCD stage, such as amyloid deposition and clinical scale, is still unclear.

In this study, we presented a novel IPLFCS analysis framework aimed at identifying SCD biomarkers. We further studied the relationship between IPLFCS biomarkers and amyloid deposition as well as neuropsychological manifestations. Importantly, to ensure the reliability of the results, we employed two cohorts from Chinese and Western medical centers. Additionally, biomarkers have better performance in disease monitoring compared to commonly used functional indicators. Finally, we enrolled patients with cognitive impairment to explore how the IPLFCS biomarkers changed throughout the AD continuum.

## 2. IPLFCS Analytical Framework

**Figure 1:**
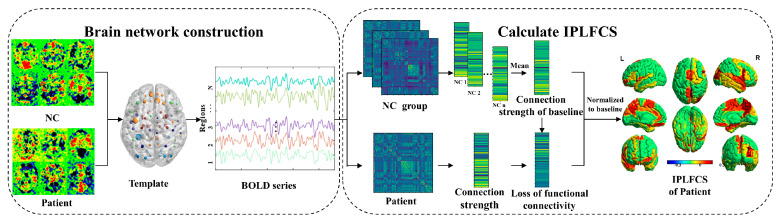
The analysis framework for IPLFCS.

The framework’s detailed flow, divided into two primary steps, is illustrated in Figure 1: brain network construction and establishment of the IPLFCS method. Specifically, the FC matrix was calculated for the participants’ preprocessed fMRI images, and the IPLFCS was calculated by normalizing the FC of the NC group.

### 2.1. Brain Network Construction

We constructed a functional brain connectivity network for each subject based on preprocessed fMRI images. First, the study employed an automatic anatomical labeling (AAL) template, consisting of 90 gray matter brain regions, to define the subject’s brain regions [34]. The time series of the corresponding brain regions was derived from the mean BOLD signals of the voxel levels. Pearson’s linear correlation analyses were performed for the time series obtained for each brain regions. This allowed us to calculate the correlation coefficients between brain regions, and a correlation matrix was constructed. Finally, to normalize the correlation coefficients, Fisher’s z-transform was utilized. The correlation coefficient is calculated as follows:

(1) $$\rho_{\left(X,Y\right)}=\frac{\sum_{i=1}^{n}\left(x_{i}-\bar{x}\right)\left(y_{i}-\bar{y}\right)}{\sqrt{\sum_{i=1}^{n}{\left(x_{i}-\bar{x}\right)}^{2}}\sqrt{\sum_{i=1}^{n}{\left(y_{i}-\bar{y}\right)}^{2}}}$$

BOLD time series of brain regions are represented using $x_{i}$ and $y_{i}$. $\bar{x}$ and $\bar{y}$ are the average values of the BOLD time-series, which is composed of n data points.

For FC, Pearson correlation coefficients between paired ROI time series were estimated, and Fisher’s z-transform was performed to quantify FC:

(2) $$FC_{\left(X,Y\right)}=0.5*\ln\frac{\left(1+\rho_{\left(X,Y\right)}\right)}{\left(1-\rho_{\left(X,Y\right)}\right)}$$

where $\rho_{\left(X,Y\right)}$ is the Pearson correlation coefficient between X and Y. For each participant, a 90 × 90 weighted adjacency FC matrix was constructed.

### 2.2. Calculation of IPLFCS

PLFCS is a novel group level functional metric. We calculated the PLFCS by determining the difference in FC between the groups and scaled the difference to the control group [32,33]. The PLFCS was calculated as follows:

(3) $$PLFCS_{x}=\frac{\sum_{y=1}^{x-1}mFCa_{y,x}-\sum_{y=1}^{x-1}mFCb_{y,x}+\sum_{y=x+1}^{n}mFCa_{y,x}-\sum_{y=x+1}^{n}mFCb_{y,x}}{\sum_{y=1}^{x-1}mFCb_{y,x}+\sum_{y=x+1}^{n}mFCb_{y,x}}$$

where $mFCa_{y,x}$ is the mean FC strength between brain region x and brain region y of the disease group, $mFCb_{y,x}$ is the mean FC matrix between x and y of the control group, n is the total number of brain regions.

To individualize these metrics, we propose an IPLFCS calculation model. The FC matrix of each subject was used as an input parameter, and the group-level FC matrix of the normal control group from cohort 1 and cohort 2 was used for normalization. The IPLFCS was the difference in mean FC between the participants and the NC group, which is scaled to the mean FC of the NC group. The difference refers to the FC of the participants subtracted by the average FC of the normal control group at the level of brain regions. Therefore, the lower the IPLFCS, the higher the loss of FC in the patient. The IPLFCS was calculated as follows:

(4) $$IPLFCS_{x}=\frac{m*\sum_{y=1}^{x-1}FCa_{y,x}-\sum_{k=1}^{m}(\sum_{y=1}^{x-1}FCb_{y,x,k})+m*\sum_{y=x+1}^{n}FCa_{y,x}-\sum_{k=1}^{m}(\sum_{y=x+1}^{n}FCb_{y,x,k})}{\sum_{k=1}^{m}(\sum_{y=1}^{x-1}FCb_{y,x,k}+\sum_{y=x+1}^{n}FCb_{y,x,k})}$$

where ${\mathrm{IPLFCS}}_{x}$ is the IPLFCS of brain region x at an individual level, n represents the total number of brain regions, the population size of the control group is denoted by m, the FC matrix of participant brain regions x and y is represented by $FCa_{y,x}$, and $FCb_{y,x,k}$ is the FC of the control group of participant k brain regions x and y.

## 3. Materials and Methods

### 3.1. Subjects

Our study consisted of two cohorts. All participants in cohort 1 were from the Sino Longitudinal Study on Cognitive Decline (SILCODE) project [35]. We included 61 SCD patients and 91 well-matched NC individuals. To verify the effectiveness of the IPLFCS in the entire spectrum of AD, we selected 44 MCI and 13 AD patients from the SILCODE project. All subjects underwent fMRI scans. Among them, 44 NC and 36 SCD subjects (cohort 1 with PET) had additional amyloid-PET (18-F [AV45]) images taken. Using an established cutoff of a priori cortical standardized uptake value ratio (SUVR) > 1.18, we identified amyloid positivity in 20 NC participants and 14 SCD participants [36,37]. The cognitive function of NC1 and SCD1 subjects was extensively assessed, using several scales. Global cognition was measured using the Mini-Mental State Examination (MMSE) scale, while subjective complaints were evaluated using the Subjective Cognitive Decline-9 (SCD-9) scale. Memory function was assessed using the Auditory Verbal Learning Test-Long Delayed Memory (AVLT-N5) and Recognition (AVLT-N7) scales, and mood was evaluated using the Hamilton Depression (HAMD) and Hamilton Anxiety Disorder (HAMA) scales [35]. The participants with MCI and AD provided demographic information and underwent comprehensive neuropsychological assessments.

We included 140 patients with NC, 75 with SCD, 205 with MCI, and 94 with AD from the Alzheimer’s Disease Neuroimaging Initiative (ADNI) database as cohort 2 to validate the effectiveness of this study across ethnic groups. All subjects underwent fMRI scans. Among them, 64 NC and 50 SCD subjects (cohort 2 with PET) had additional amyloid-PET (18-F [AV45]) images taken, with those positive for amyloid including 29.7% of NC (*n* = 19) and 38% of SCD (*n* = 19). From the ADNI database, demographic characteristics and neurocognitive performance data were obtained (Figure S1).

### 3.2. Inclusion Criterion

All participants in the SILCODE project were Mandarin-speaking and right-handed. The NC participants were volunteers whose scores were within the normal range on neuropsychological tests and did not report any concerns about cognitive decline. To determine the inclusion criteria for SCD participants, we adopted the conceptual framework proposed in 2014 [3], in combination with relevant references from our previous studies [1,35]. The details were as follows: (a) the cognitive decline that is persistent self-perceived is not related to the presence of acute events, (b) concerns (worries) associated with memory complaints, and (c) performance on all clinical scales. The principal basis for MCI diagnosis is the neuropsychological approach [38]. The guidelines of the NIA-AA working group were used as diagnostic criteria for AD [39]. The exclusion criteria for study participants included a history of stroke, syphilis infection, brain damage, or other medical conditions [35]. To ensure accuracy, two experienced neurologists were solicited to examine the diagnosis. The diagnosis of participants in cohort 2 was carried out using the standard criteria as outlined in the ADNI procedures manual, the details of which can be found at http://www.adni-info.org.

### 3.3. Standard Protocol Approvals, Registrations, and Patient Consents

All participants or their informants/caregivers in this study gave permission for their anonymous clinical details to be published and provided written informed consent and written consent.

### 3.4. Image Acquisition Protocol

In cohort 1, all participants underwent scanning using the same image-acquisition protocols. The imaging data were collected with 3.0 T TOF PET/MR (GE Healthcare, Milwaukee, Wisconsin, USA). The acquisition parameters for fMRI were as follows: data matrix = 64 × 64, repetition time (TR) = 2000 ms, echo time (TE) = 30 ms, slice thickness = 4.0 mm, slice number = 28, voxel size = 3.5 × 3.5 × 4 mm^3^. The following parameters were used for the MRI: TR = 6.9 ms, TE = 2.98 ms, SPGR sequence, matrix = 256 × 256, slice thickness = 1 mm, slice number = 192, inversion time (TI) = 450 ms, voxel size = 1 × 1 × 1 mm^3^.

A dynamic scan lasting for 35 min was performed for [^18^F] AV45 PET, approximately 40 min after administering an intravenous injection of 7–10 mCi [^18^F] florbetapir. The following parameters were used for PET data acquisition: 8 iterations, 32 subset matrices = 192 × 192, half-width height = 3, FOV = 350 × 350.

The fMRI images in cohort 2 were acquired using 3.0-T scanner from SIEMENS. The following were the acquisition parameters for the fMRI data: TR = 3000 ms, TE = 30 ms, number of volumes = 197, slice number = 48, voxel size = 3.44 × 3.44 × 3.40 mm^3^, slice thickness = 3.4 mm. The MRI parameters were as follows: TR = 2300 ms, TE = 2.98 ms, slice thickness = 1 mm, TI = 900 ms, voxel size = 1 × 1 × 1 mm^3^. The parameters for 18-F [AV45] PET were as follows: Number of Columns = 336.0 pixels; Number of Rows = 336.0 pixels; Number of Slices = 109.0; Slice Thickness = 2.0 mm.

### 3.5. Image Pre-Processing

In this study, the fMRI data were preprocessed in MATLAB R2016b based on DPARSF (Data Processing Assistant for Resting-State Fmri, http://rfmri.org/dpabi (accessed on 3 April 2023)). Stabilization of the initial signal by discarding the first 10 volumes. Correction for the remaining volume was completed based on the acquisition time. Then, it was realigned to the first volume. Regression was performed using the average signal extracted from the white matter (WM), cerebrospinal fluid (CSF), and Friston-24 head motion parameters. Individual average fMRI images were registered to their respective MRI scans. The deformation field space was segmented from the MRI images and normalized to the Montreal Neurological Institute (MNI) space. fMRI data underwent correction for linear drift and band-pass filtered (0.01–0.08 Hz). Lastly, a half-maximum filter was used to smooth the fMRI data.

Preprocessing of the PET data based on the Statistical Parametric Mapping 12 (SPM12; https://www.fil.ion.ucl.ac.uk/spm/software/spm12 (accessed on 3 April 2023)) package. First of all, PET images were aligned with the MRI. Segmentation was performed on T1 images to obtain tissue probability maps of gray matter (GM), WM, and CSF. Registration of the GM maps based on MNI stereotaxic template. The aligned PET images were also normalized to the MNI template. Lastly, a half-maximum (EWHM) filter was used to smooth the normalized PET images. The mean florbetapir SUVR values were calculated, and the reference region was the whole cerebellum.

### 3.6. Statistical Analysis

Continuous variables were analyzed for group differences using a two-sample t-test, while categorical variables were analyzed using the χ2 test. All statistical analyses were considered to be statistically significant if *p*-values are less than 0.05.

#### 3.6.1. Comparison between FC and IPLFCS

In addition, to compare the NC1 and SCD1 groups, two-sample t-tests were performed using the FC and IPLFCS. The average FC of the brain region and the remaining brain regions was calculated, and a vector was built for analysis. The results underwent post hoc testing correction (false discovery rate correction) to verify whether IPLFCS could detect more differences in brain regions than FC.

#### 3.6.2. Identification of IPLFCS Biomarkers for SCD

To determine robust IPLFCS biomarkers, two-sample t-tests were performed in cohort 1 with PET and cohort 1 without PET to analyze differences. The post hoc test (false discovery rate correction) was also performed on the results. We regarded the brain regions that passed the post-test in both cohorts as potential biomarkers of SCD. Similarly, to validate the effectiveness of IPLFCS biomarkers across human populations, differential brain regions were calculated in cohort 2 using the same method. The IPLFCS biomarkers discovered in different cohorts were visualized in the AD continuum using box plots.

#### 3.6.3. Correlation between IPLFCS and FC Strength

To verify the effectiveness of the IPLFCS, we first examined the Pearson correlation between the average IPLFCS and average FC strength of 90 brain regions in the SCD (SCD1, SCD2), MCI (MCI1, MCI2), and AD (AD1, AD2) groups. We hypothesized that the lower the connection strength of the disease groups, the lower the IPLFCS. Furthermore, we conducted Granger causality analysis to investigate the relationship between IPLFCS and FC within the disease group.

#### 3.6.4. Correlation Analysis of Amyloid SUVR and IPLFCS

To explore whether correlations exist between IPLFCS biomarkers and amyloid deposition, we performed Pearson correlation test between global SUVR, temporal SUVR, SUVR of biomarkers, and IPLFCS of biomarkers in cohort 1 with PET and cohort 2 with PET (NC+SCD).

#### 3.6.5. Correlation Analysis of Clinical Scale and IPLFCS

Finally, we tested the correlations coefficients between IPLFCS biomarkers and neuropsychological scales in the SCD1 group.

#### 3.6.6. Receiver Operating Characteristic Curve

The ability of potential biomarkers to distinguish between different groups was assessed using receiver operating characteristic (ROC) curves. The following groups were included: NC1 vs. SCD1, NC1 vs. MCI1, NC1 vs. AD1, NC2 vs. SCD2, NC2 vs. MCI2, NC2 vs. AD2. To evaluate the performance of the ROC curves, we calculated the areas under curves (AUCs) and also calculated their corresponding 95% confidence intervals. We obtained ROC curves for the IPLFCS of the potential biomarker and FC. In addition, calculations of regional homogeneity (ReHo) and amplitude of low-frequency fluctuations (ALFF) were performed based on the AAL template. These metrics, based on all 90 regions of the AAL, were used as comparison tests for the ROC curves.

## 4. Results

### 4.1. Demographic Findings

Detailed demographic and clinical characteristics are presented in Table 1. In Cohort 1, the SCD-9 (*p* < 0.001), HAMD scores (*p* = 0.044) and HAMA (*p* = 0.002) of the SCD group were higher than in the NC group. Additionally, there was a sex imbalance (*p* = 0.004). There were no significant differences in age, educational level, and other cognitive performances. In cohort 2, the NC group had a lower educational level (*p* = 0.035) than the SCD group, but there were no differences in age, gender, and cognitive performance.

### 4.2. Comparison between FC and IPLFCS

**Figure 2:**
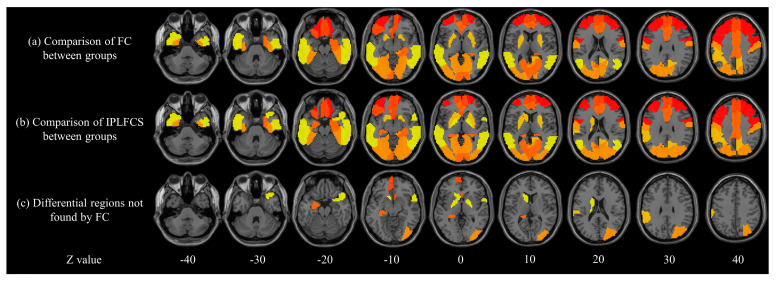
Group comparison between NC and SCD in brain regions. (**a**) Comparison of FC between group; (**b**) Comparison of IPLFCs between group; (**c**) Differential brain regions were found by the between-group comparison of IPLFCS but not in the between-group comparison of FC. Z value represents the *z*-axis of standard space. Differential brain regions found: amygdala and temporal gyrus (see Z = −20), hippocampus and olfactory (see Z = −10), superior frontal gyrus and lenticular nucleus (see Z = 0), caudate nucleus (see Z = 10), occipital gyrus (see Z = 20), and supramarginal gyrus (see Z = 30).

Figure 2 shows that the IPLFCS could identify more different brain regions in the between-group comparison of NC and SCD. Figure 2a,b show the between-group comparison of FC and IPLFCS, respectively, at the level of the brain regions. Figure 2c shows that the IPLFCS found more differential brain regions, which include the olfactory cortex, superior frontal gyrus, hippocampus, amygdala, lenticular nucleus, occipital gyrus, supramarginal gyrus, caudate nucleus, and temporal gyrus.

### 4.3. IPLFCS Biomarkers for SCD

**Figure 3:**
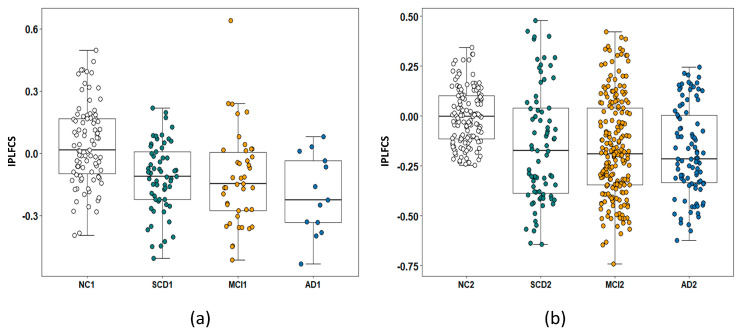
The IPLFCS in AD continuum. (**a**) The IPLFCS of LMTG in groups of cohort 1. (**b**) The IPLFCS of LMTG in groups of cohort 2.

In cohort 1 with PET and cohort 1 without PET, we conducted an analysis of the difference in IPLFCS in 90 brain regions using a two-sample t-test. Only the left middle temporal gyrus (LMTG) passed the post hoc testing corrections for brain regions. Specifically, the post hoc testing corrections used the Benjamini–Hochberg method for FDR correction. We found that the LMTG also passed post hoc testing corrections in Cohort 2. Supplementary Table S1 shows all brain regions with significant differences. The results in Figure 3 demonstrated that the IPLFCS of the LMTG showed an obvious downward trend in both the cohorts.

### 4.4. Correlation between IPLFCS and FC

**Figure 4:**
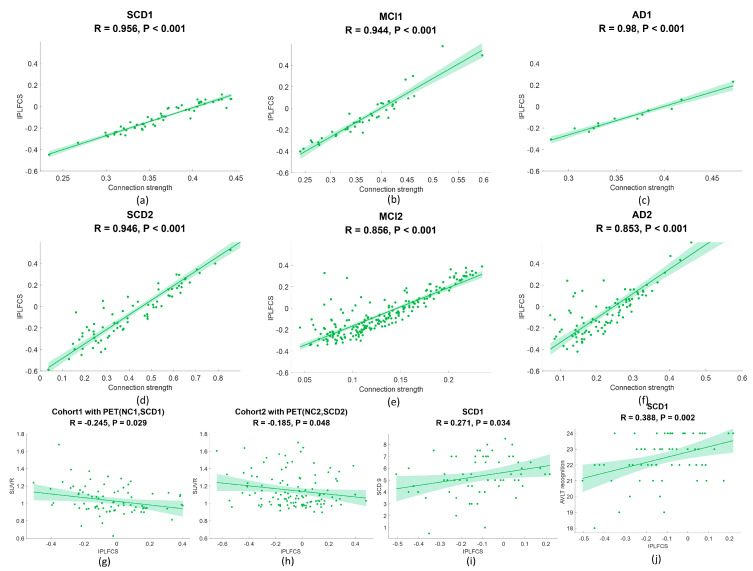
Linearities between IPLFCS and SUVR and clinical scores in different groups. (**a**) Correlation analysis between IPLFCS and FC in the SCD1. (**b**) Correlation analysis between IPLFCS and FC in the MCI1. (**c**) Correlation analysis between IPLFCS and FC in the AD1. (**d**) Correlation analysis between IPLFCS and FC in the SCD2. (**e**) Correlation analysis between IPLFCS and FC in the MCI2. (**f**) Correlation analysis between IPLFCS and FC in the AD2. (**g**) Correlation analysis between IPLFCS and SUVR of LMTG in Cohort 1 with PET. (**h**) Correlation analysis between IPLFCS and SUVR of LMTG in Cohort 2 with PET. (**i**) Correlation analysis between IPLFCS of LMTG and SCD-9 scores in the SCD1 group. (**j**) Correlation analysis between IPLFCS of LMTG and AVLT recognition scores in the SCD1 group.

Figure 4 shows the correlations between average FC strength and average IPLFCS in the SCD1, MCI1, AD1, SCD2, MCI2, and AD2 groups. In different disease groups, the results demonstrated that the correlations between FC strength and IPLFCS were strongly significant (*p* < 0.001) in SCD1 (r = 0.956, Figure 4a), MCI1 (r = 0.944, Figure 4b), AD1 (r = 0.980, Figure 4c), SCD2 (r = 0.946, Figure 4d), MCI2 (r = 0.856, Figure 4e), and AD2 (r = 0.853, Figure 4f). It was noted the IPLFCS and FC strength were positively correlated, indicating that the IPLFCS could perfectly characterize FC strength. Supplementary Table S2 provides the results of the Granger causality analysis for the average FC strength and average IPLFCS. No causal relationship was found between the average FC strength and average IPLFCS in SCD1, MCI1, AD1, SCD2, MCI2, and AD2 groups. While we found a correlation between IPLFCS and FC, we did not observe a direct causal relationship. We conclude that these two features are interrelated, but they are not causally related.

### 4.5. Correlation Analysis of Amyloid SUVR and IPLFCS

A weak negative correlation was found between the amyloid SUVR of the LMTG and the corresponding IPLFCS in cohort 1 with PET (r = −0.245, *p* = 0.029, Figure 4g). We also found significant correlations between the IPLFCS of the LMTG and important brain regions, including the hippocampus and temporal regions. No significant correlation was found between the whole-brain SUVR and IPLFCS. Supplementary Table S3 provides a detailed overview of the results. Figure 4h shows a negative correlation between the IPLFCS of the LMTG and the corresponding SUVR in cohort 2 with PET (r = −0.185, *p* = 0.048).

### 4.6. Correlation Analysis of Clinical Scale and IPLFCS

Correlation analysis showed that the SCD 9 scale (r = 0.271, *p* = 0.034, Figure 4i) as well as AVLT identification scale (r = 0.388, *p* = 0.002, Figure 4j) were positively correlated with IPLFCS in the SCD1 group.

### 4.7. Receiver Operating Characteristic Curve

**Figure 5:**
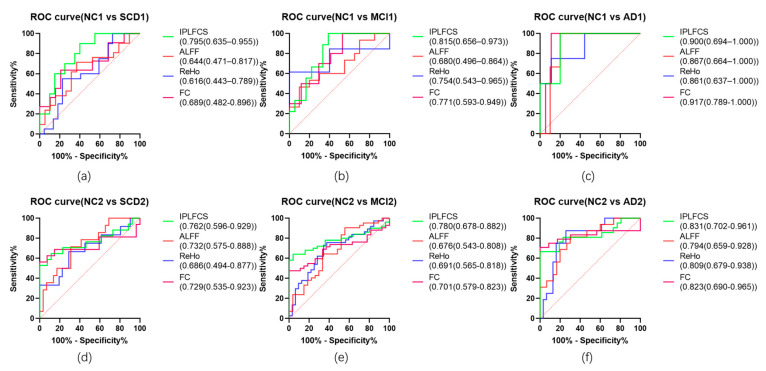
The results of ROC analysis among IPLFCS, ALFF, ReHo and FC. (**a**) The ROC results of NC1 vs. SCD1. (**b**) The ROC results of NC1 vs. MCI1. (**c**) The ROC results of NC1 vs. AD1. (**d**) The ROC results of NC2 vs. SCD2. (**e**) The ROC results of NC2 vs. MCI2. (**f**) The ROC results of NC2 vs. AD2.

Figure 5 displays the results of the ROC analysis. The IPLFCS achieved the highest AUCs (0.795 (0.635–0.955)) in distinguishing NC from SCD when compared with ALFF (0.644 (0.471–0.817)), ReHo (0.616 (0.443–0.789)), and FC (0.689 (0.482–0.896)). In distinguishing NC1 from MCI1, the AUC of the IPLFCS reached 0.815 (0.656–0.973), whereas the AUCs of the ALFF, ReHo, and FC were 0.680 (0.496–0.864), 0.754 (0.543–0.965), and 0.771 (0.593–0.949). In distinguishing NC1 from AD1, the IPLFCS (0.900 (0.694–1.000)), ALFF (0.867 (0.664–1.000)), ReHo (0.861 (0.637–1.000)), and FC (0.917 (0.789–1.000)) all showed good classification performance.

In distinguishing NC2 from SCD2, the AUC of the IPLFCS was 0.762 (0.596–0.929), whereas the AUCs of ALFF, ReHo, and FC were 0.732 (0.575–0.888), 0.686 (0.494–0.877), and 0.729 (0.535–0.923). In distinguishing NC2 from MCI2, the AUC of the IPLFCS was 0.780 (0.678–0.882), whereas the AUCs of ALFF, ReHo, and FC were 0.676 (0.543–0.808), 0.691 (0.565–0.818), and 0.701 (0.579–0.823). In distinguishing NC2 from AD2, the IPLFCS (0.831 (0.702–0.961)), ALFF (0.794 (0.659–0.928)), ReHo (0.809 (0.679–0.938)), and FC (0.823 (0.690–0.965)) performed well.

## 5. Discussion

In this study, we found that the IPLFCS could detect more SCD-related areas than traditional FC. Thus, the IPLFCS of the LMTG may be a potential biomarker of SCD. Notably, we employed methodological optimization to ensure the robustness of our results. First, we performed multiple permutation tests on data from the two cohorts. These measures reinforce the power of the results. We further demonstrated that the IPLFCS of the LMTG presented a gradually deteriorating trend in the three groups of SCD, MCI and AD. It also showed significant correlations with amyloid deposition and cognitive performance. Therefore, the IPLFCS of the LMTG is likely to be a better indicator for the inclusion of SCD, which might increase the monitoring capabilities via objective biomarkers and decrease the errors of subjective descriptions to a certain extent. Monitoring SCD can help researchers understand the occurrence and progression of SCD, promoting knowledge and treatment of this disease.

Currently, potential biomarkers of the preclinical AD spectrum using different neuroimaging modalities have been explored [39,40,41,42]. We noted that the diagnostic efficiency of the functional features of MRI was superior to that of the structural or metabolic modalities, indicating that it could be a sensitive and powerful biomarker for SCD [10,11]. The IPLFCS is a novel FC metric that standardizes the connectivity strength of the baseline and better addresses the issue of high noise in fMRI compared to FC. Our results showed that IPLFCS could identify more differential brain regions in SCD populations than the conventional FC. Therefore, we further performed the individually specified IPLFCS to characterize the quantitative decrease degree. The IPLFCS of the LMTG was found to have a robust diagnostic efficacy, with accuracy rates of 80% and 76% in categorizing SCD from NC in the different cohorts, respectively. Our result of a single modality also obtained a good classification performance similar to that of the multimodal analysis, which will contribute to reducing the medical cost of patients. Evidence indicated that the IPLFCS of the LMTG exhibited increased diagnostic specificity for SCD in practical applications.

As individuals age and experience memory deficits, a reduction in FC is evident and is linked to factors such as the massive loss of neurons and synapses during the development of aging and AD [43]. However, conventional neuroimaging biomarkers are difficult to distinguish due to subtle reductions in connection strength in the early stages of SCD [44,45]. Therefore, the establishment of reliable and sensitive markers for early diagnosis and monitoring of brain connectivity remains an important challenge. In this study, significant differences in IPLFCS were discovered between the independent NC and SCD groups. Additionally, the IPLFCS of the LMTG progressively decreased across the AD continuum. Correlation analysis demonstrated that the IPLFCS in this region was related to cognitive scores. These results suggest that functional loss of the LMTG occurs as early as the SCD stage, with progressive progression of the IPLFCS and concomitant cognitive decline. The LMTG is a crucial region in the processing of memory [46,47,48]. It has FC to the hippocampus as well as different parcellations in the frontal and parietal. It is responsible for rapid encoding of new associations and memory consolidation [49,50]. An fMRI study reported that the LMTG is involved in sublexical processing of Chinese character recognition [51]. The FC patterns revealed that the middle temporal gyrus exhibited strong connections with the frontal-parietal language areas and was mainly associated with the default mode network, as well as in sound recognition. The FC patterns of LMTG may improve or remain stable with age in neurodegenerative diseases, to meet the cognitive needs of the elderly [52]. If the FC pattern of LMTG weakens in elderly individuals, it could result in poorer performance on memory encoding and recognition tasks [53,54]. Therefore, by assessing the functional status of the LMTG, a better understanding of the mechanisms underlying SCD can be obtained, and it may help in the prevention and treatment of cognitive disorders. Last but not least, this study found that the IPLFCS of the LMTG was related to amyloid deposition. These pathologies cause neuronal loss, synaptic connection disruption, and deficits in brain function.

## 6. Limitations

While this study has identified a stable biomarker, there are still several limitations that need to be addressed. One of the primary limitations is the small number of SCD individuals with amyloid deposition included in the study. In the future, it should be considered to increase the sample size. Second, we studied the relationship between amyloid deposition and IPLFCS. It should be considered to analyze the deposition patterns of tau as a future research direction to follow up on the current study. The study of tau protein could be helpful in explaining the molecular mechanism of IPLFCS. Third, we only conducted a cross-sectional study among individuals with NC, SCD, MCI, and AD. However, longitudinal research is lacking. In subsequent studies, follow-up observational studies on patients will help verify our results. We acknowledge the limitation of our study that the participants had mismatched gender and educational backgrounds, which may have introduced confounding variables. We will consider using more rigorous inclusion criteria to ensure better matching of gender and educational backgrounds.

## 7. Conclusions

In summary, this study demonstrated that IPLFCS of the LMTG may serve as a potential biomarker of SCD. Furthermore, it gradually worsened across the AD continuum, which suggests that the reduced functionality of the LMTG during the SCD stage could potentially signify cognitive decline in the future. This can be an indicator for the progression.

## Figures and Tables

**Table 1:** Demographic characteristics.

	Cohort 1					Cohort 2				
	NC (91)	SCD (61)	MCI (44)	AD (13)	*p* Value	NC (140)	SCD (75)	MCI (205)	AD (94)	*p* Value
Age (years)	65.5 ± 5.7	66.7 ± 5.9	66.9 ± 8.4	71.4 ± 8.9	0.204 ^b^	72.0 ± 5.9	71.6 ± 5.5	71.3 ± 7.2	74.0 ± 7.8	0.560 ^b^
Education (years)	12.5 ± 3.0	12.8 ± 2.7	10.6 ± 3.9	11.5 ± 4.2	0.488 ^b^	16.2 ± 2.2	16.9 ± 2.5	16.0 ± 2.7	14.9 ± 2.5	0.035 ^b,^*
Gender (male/female)	38/53	12/49	19/25	3/10	0.004 ^a,^*	59/81	32/43	100/105	45/49	0.941 ^a^
Aβ(+/−)	24/20	22/14	-	-	0.555 ^a^	45/19	31/19	-	-	0.350 ^a^
HAMD	2.8 ± 3.8	5.3 ± 1.7	6.4 ± 6.8	4.7 ± 5.7	0.044 ^b,^*	-	-	-	-	-
HAMA	3.3 ± 2.2	5.3 ± 4.5	5.0 ± 4.9	4.5 ± 4.4	0.002 ^b,^*	-	-	-	-	-
SCD-9	3.5 ± 2.2	5.3 ± 1.7	2.0 ± 1.8	2.8 ± 0.8	<0.001 ^b,^*	-	-	-	-	-
MMSE	28.9 ± 1.4	28.9 ± 1.3	26.0 ± 2.7	17.0 ± 5.3	0.861 ^b^	28.7 ± 4.6	29.0 ± 0.9	27.3 ± 2.5	21.1 ± 3.5	0.119 ^b^
AVLT-N5	7.5 ± 1	7.1 ± 1.9	3.8 ± 2.4	0.9 ± 0.4	0.248 ^b^	-	-	-	-	-
AVLT- Recognition	22.4 ± 1.5	22.4 ± 1.4	19.7 ± 2.6	15.7 ± 2.6	0.881 ^b^	-	-	-	-	-

Discrete variables are given as mean ± standard deviation. * denotes a significant difference. ^a^ the *p* value was obtained by χ2 test; ^b^ the *p* value was obtained by two-sample t-tests.

## Data Availability

The datasets generated and/or analyzed during the current study are not publicly available but are available from the corresponding author on reasonable request.

## Supplementary Materials

The following supporting information can be downloaded at: https://www.mdpi.com/article/10.3390/biology12040564/s1, Figure S1: Workflow show ADNI cohort participant selection details in (a) NC, (b) SCD, (c) MCI and (d) AD. Abbreviations: NC, normal control; SCD, subjective cognitive decline; MCI, mild cognitive im-pairment; AD, Alzheimer’s disease; Table S1: The ROI-based brain regions with significant differences; Table S2: Results of causal analysis; Table S3: Correlation analysis of SUVR and proportional loss.
