# Supplementary material for: Individual Proportion Loss of Functional Connectivity Strength: A Novel Individual Functional Connectivity Biomarker for Subjective Cognitive Decline Populations
Source: Biology (Basel). 2023 Apr 7;12(4):564. doi: 10.3390/biology12040564 (PMC10135935; doi:10.3390/biology12040564)
Supplement: Supplementary file 1 [file biology-12-00564-s001.zip › biology-2247116-supplementary.pdf]

Supplementary Table S1. The ROI-based brain regions with significant differences.

The ROIs with significant differences(AAL template)					
Cohort 1 with PET (NC VS SCD)			Cohort 2 (NC VS SCD)		
Regions	Anatomical classification	P value (corrected)	Regions	Anatomical classification	P value (corrected)
Superior frontal gyrus, medial orbital(left)	Prefontal	0.041	Middle temporal gyrus(left)	Temporal	<0.001
Middle temporal gyrus(left)	Temporal	0.021	Middle temporal gyrus(right)	Temporal	<0.001
Cohort 1 without PET (NC VS SCD)					
Middle frontal gyrus(left)	Prefontal	0.026			
Middle frontal gyrus(right)	Prefontal	0.026			
Calcarine fissure and surrounding cortex(right)	Occipital	0.031			
Superior occipital gyrus(left)	Occipital	0.035			
Middle temporal gyrus(left)	Temporal	0.026			

Abbreviations: SCD, subjective cognitive decline; NC, normal control; ROI, region of interest; AAL, anatomical automatic labeling.

Supplementary Table S2. Results of causal analysis.

	SCD 1	MCI 1	AD 1	SCD 2	MCI 2	AD 2
P value	0.845	0.878	0.942	0.782	0.474	0.101

Abbreviations: SCD, subjective cognitive decline; MCI, mild cognitive impairment; AD, Alzheimer's disease.

Supplementary Table S3. Correlation analysis of SUVR and proportional loss.

The ROIs with significant differences(AAL template)<br>Cohort 1 with PET (NC VS SCD)			
Regions	Anatomical classification	R	P value
Whole brain		-0.176	0.119
Hippocampus_L	Temporal	-0.259	0.020
Hippocampus_R	Temporal	-0.281	0.012
ParaHippocampal_L	Temporal	-0.165	0.143
ParaHippocampal_R	Temporal	-0.227	0.043
Amygdala_L	Temporal	0.142	0.207
Amygdala_R	Temporal	-0.202	0.073
Fusiform_L	Temporal	-0.329	0.003
Fusiform_R	Temporal	-0.299	0.007
Heschl_L	Temporal	-0.173	0.126
Heschl_R	Temporal	-0.216	0.054
Temporal_Sup_L	Temporal	-0.201	0.074
Temporal_Sup_R	Temporal	-0.196	0.082
Temporal_Pole_Sup_L	Temporal	-0.072	0.526
Temporal_Pole_Sup_R	Temporal	0.209	0.063
Temporal_Mid_L	Temporal	-0.245	0.029
Temporal_Mid_R	Temporal	-0.200	0.075
Temporal_Pole_Mid_L	Temporal	0.059	0.606
Temporal_Pole_Mid_R	Temporal	0.053	0.640
Temporal_Inf_L	Temporal	-0.217	0.053
Temporal_Inf_R	Temporal	-0.123	0.275

Abbreviations: SCD, subjective cognitive decline; NC, normal control; ROI, region of interest; AAL, anatomical automatic labeling.

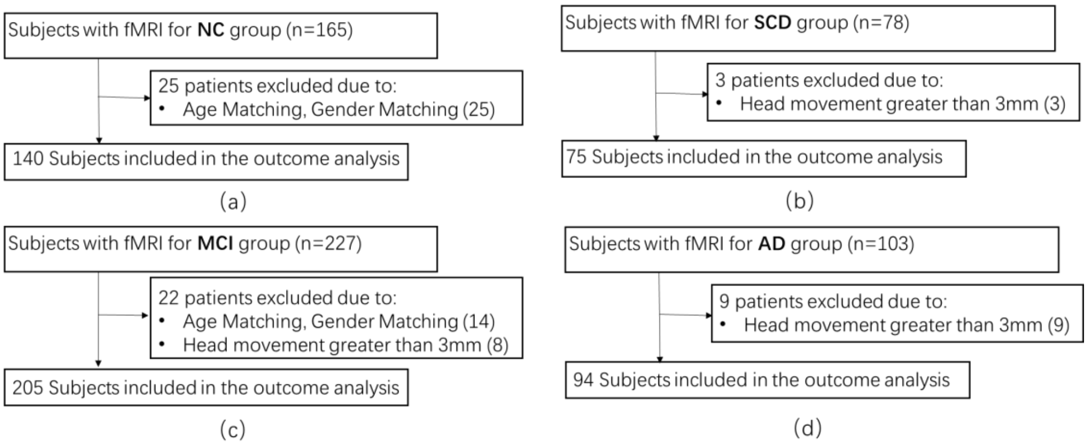

Supplementary Figure S1. Workflow show ADNI cohort participant selection details in (a) NC, (b) SCD, (c) MCI and (d) AD. Abbreviations: NC, normal control; SCD, subjective cognitive decline; MCI, mild cognitive impairment; AD, Alzheimer's disease.
